# Standardized ileal digestibility of amino acids in broiler chickens fed single or mixture of feed ingredients-based diets with or without *Eimeria* challenge

**DOI:** 10.1016/j.psj.2022.101839

**Published:** 2022-03-10

**Authors:** Emily Kim, John R. Barta, William Lambert, Elijah G. Kiarie

**Affiliations:** ⁎ Department of Animal Biosciences, University of Guelph, Guelph, ON, Canada; † Department of Pathobiology, University of Guelph, Guelph, ON, Canada; ‡ METEX NØØVISTAGO, Paris, France

**Keywords:** amino acids, endogenous amino acid losses, standardized ileal digestibility of amino acids, coccidiosis, broiler chickens

## Abstract

The effect of *Eimeria* challenge on standardized ileal digestibility (**SID**) of amino acids (**AA**) in major poultry feed ingredients fed to broiler chickens was determined. A total of 840 male 9-day-old Ross 708 chicks were placed in 84 cages (10 birds/cage) and allocated to either a nitrogen-free diet (**NFD**) or one of the 6 test diets based on a single or mixture of feed ingredients as the sole source of AA (n = 12). Test diets were: 1) corn, 2) wheat, 3) soybean meal (**SBM**), 4) pork meal (**PM**), 5) corn, SBM, and PM (**CSP**), and 6) wheat, SBM, and PM (**WSP**). On d 10, birds in 6 cages/diet were orally gavaged with 1 mL of *E. acervulina* and *E. maxima* mixture and the other 6 cages with sham. On d 15, birds were bled for plasma AA and necropsied for intestinal lesion scores and ileal digesta samples. Challenge decreased (*P* < 0.05) plasma concentration of Arg, His, Thr, Asp, Gln, and Tyr and increased concentration of Lys, Ile, Leu, and Val. There was a diet by challenge interaction (*P* < 0.05) on intestinal lesion scores with birds fed mixed diets exhibiting more severe lesions than birds fed single ingredient diets. Diet by challenge interacted (*P* < 0.05) on ileal total endogenous flow (**ITEF**) of AA except for Arg, Met, Ala, Asp, and Cys, such that challenged birds fed the mixed, particularly WSP, had higher ITEF of AA compared to single ingredients birds. Diet and challenge interaction (*P* < 0.05) was observed for SID of Arg, Thr, Val, Glu, and Gly. Challenge decreased (*P* < 0.05) SID of most AA except for Met, Asp, and Cys with the largest impact seen on Lys, His, Ser, and Thr. With exception of Arg, Thr, Asp, and Cys, birds fed mixed diets had higher (*P* < 0.05) SID values compared to birds fed single ingredients. In conclusion, *Eimeria* reduced plasma availability and ileal digestibility of most AA. However, challenge interaction with diet composition on SID of some AA warrants further investigations.

## INTRODUCTION

Feed constitutes more than half the cost of broiler production, with a considerable proportion of this cost allotted towards provision of energy and amino acids (**AA**) to meet the requirements for maintenance and production (Adedokun et al., 2016). Arguably, adoption of the ideal protein, and standardized ileal digestibility (**SID**) of AA concepts along with commercial availability of feed-grade AA, has enabled poultry nutritionists to formulate cost-effective and optimal diets for poultry (Adedokun et al., 2012, 2016). However, although formulating on SID basis produces a better defined performance target, central to this is a functional gastrointestinal tract (**GIT**) to digest and absorb nutrients and to excrete waste products (Kiarie et al., 2013). A functional gut is a result of the status of many interrelated elements such as villi architecture, gut-associated immune system and microbiome (Kiarie et al., 2013). However, disruption of this system by enteric pathogens, whether producing clinical disease or not, interferes with the nutrient supply and requirement balance (Kiarie et al., 2019). *Eimeria*, the causative agent for coccidiosis, is ubiquitous in poultry production operations and causes significant economic losses due to increased mortality and morbidity, and poor growth performance (Allen and Fetterer, 2002; Teng et al., 2021). The reduced feed intake and feed efficiency commonly seen in birds afflicted by coccidiosis can be attributed to the damage of the intestinal epithelium, consequently resulting in poor nutrient absorption (Persia et al., 2006; Adedokun and Adeola, 2016; Kim et al., 2017; Kiarie et al., 2019).

Although the use of anticoccidials has successfully controlled and decreased the incidence of coccidiosis in poultry production for decades, long-term and large-scale use of these products have led to concerns over the development of resistance (Peek and Landman, 2011). Alternatively, the use of live attenuated or nonattenuated vaccines to stimulate the immune system to produce antibodies against *Eimeria* antigens has been shown to be quite successful (Williams, 2002). However, the nature of vaccines is to mimic their natural virulence through the exposure of controlled doses (Williams, 2002; Price, 2012). Thus, the vaccine strains still possess the ability to undergo their replicative life cycle in the host intestines and cause varying levels of tissue damage, which may negatively affect nutrient digestibility and weight gain (Gautier and Rochell, 2020).

It is standard practice to use SID values of individual ingredients for accurate feed formulation (Adedokun et al., 2012, 2016). However, the industry relies on compiled databases (e.g., National Research Council, INRA-CIRAD-AFZ Feed tables, Brazilian tables, Evonik Animal Nutrition, Dutch CVB feed tables, and Premier Atlas among others) for the coefficients of SID of AA for feed ingredients. Arguably, these coefficients are derived by correcting for basal endogenous AA losses in birds free of enteric disturbances. *Eimeria*-induced mucogenesis and enterocyte turnover, as well as postabsorptive metabolic changes and immune system activation, likely influence endogenous AA losses (Adedokun et al., 2012, 2016). While previous research has demonstrated the negative impact of a coccidiosis challenge and vaccination on AA digestibility, such studies primarily reported apparent ileal digestibility of AA in complete feed (e.g., as recently reviewed, Kim et al., 2022). Feed ingredients are inherently unique in terms of antinutritional factors and indigestible components (Adedokun et al., 2011, Bryan, 2019, Kiarie, 2021). For this reason, AA digestibility varies from one feedstuff to another. As such, it would be necessary to characterize the dynamics of AA digestibility under a coccidiosis challenge in different feed ingredients (Gautier and Rochell, 2020). Therefore, the objective of this study was to determine SID of AA in poultry feed fed as single ingredient or mixture of ingredients to broiler chicks subjected to an *Eimeria* challenge.

## MATERIALS AND METHODS

The experimental protocol (#3521) was reviewed and approved by the University of Guelph Animal Care Committee and birds were cared for in accordance with the Canadian Council on Animal Care guidelines (2009).

### Birds and Housing

A total of 840 male day-old (Ross 708) chicks were procured from a local hatchery (Maple Leaf Foods, New Hamburg, ON, Canada) and placed in 84 cages (10 birds/cage). The cages (each measuring 20″ × 30″; Ford Dickison Inc., Mitchell, ON, Canada) were housed in an environmentally controlled room. The room had a total of 96 cages installed in 2 rows separated by a 36″ walkway and cages stacked in 2 tiers of 24 cages each side. The room temperature was set at 32°C on d 0 and gradually brought down to 27°C by d 14. The lighting program was 23 h of light (20+ LUX) from d 0, gradually decreasing to 18 h (20+ LUX) on d 3 and followed by 16 h of light (10–15 LUX) from d 4 onward. The cages were equipped with feeders and water drinking nipples.

### Dietary Treatments

A basal diet was formulated to meet breeder (Ross 708) nutrient specifications (Table 1) for rearing birds prior to feeding test diets. Six test diets were formulated to contain a single feed ingredient or a mixture of feed ingredients as the sole source of AA. The test diets were: 1) corn, 2) wheat, 3) soybean meal (**SBM**), 4) pork meal (PM), 5) corn + soybean meal + pork meal (**CSP**), 6) wheat + soybean meal + pork meal (**WSP**). A nitrogen-free diet (**NFD**) was also formulated for estimating ileal endogenous AA losses. All diets were prepared in mash form and, with exception of basal starter diet, contained 0.3% titanium dioxide as the indigestible marker (Table 1). Due to the differences in composition of ingredients, crude protein and energy contents varied among diets, however, was kept similar between grain diets (corn and wheat), protein diets (soybean meal and pork meal), and in the mixed diets. Diets were fortified with minerals and vitamins to meet or exceed Ross 708 requirements (Aviagen, 2019).

**Table 1:** Composition of the starter and experimental diets, as fed basis.

Item	Starter	NFD^2^	Corn	Wheat	SBM^3^	PM^4^	CSP^5^	WSP^6^
Ingredient (%)								
Corn	51.89	-	85.04	-	-	-	50.00	-
Soybean meal	39.15	-		-	43.37	-	30.00	30.00
Wheat	-	-	-	60.00	-	-	-	35.28
Pork meal	-	-				34.76	3.00	3.00
Soy oil	3.48	2.71	0.28	1.03	1.57	1.73	0.41	0.85
Monocalcium phosphate	2.13	2.35	2.21	2.12	1.93	-	1.25	1.20
Limestone,	1.12	1.25	1.05	1.29	1.11	-	0.57	0.71
Vitamins and trace minerals premix^1^	1.00	1.00	1.00	1.00	1.00	1.00	1.00	1.00
Salt	0.27	0.26	0.22	0.20	0.26	0.06	0.23	0.22
Sodium bicarbonate	0.13	0.20	0.23	0.20	0.16	-	0.13	0.11
L-Lysine-HCL	0.24	-	-	-	-	-	-	-
DL Methionine	0.48	-	-	-	-	-	-	-
L-Threonine	0.12	-	-	-	-	-	-	-
Cornstarch	-	77.22	7.93	29.41	44.94	49.50	11.75	24.38
Dextrose	-	8.93	0.92	3.40	5.20	5.72	1.36	2.82
Cellulose	-	5.00	-	-	-	5.00	-	-
Choline chloride	-	-	-	-	-	0.32	-	-
Magnesium oxide	-	0.07	-	-	-	0.10	-	-
Potassium carbonate	-	0.72	0.82	1.05	0.16	1.50	-	0.14
Titanium dioxide	-	0.30	0.30	0.30	0.30	0.30	0.30	0.30
Calculated provisions	-	-	-	-	-	-	-	-
AME (kcal/kg)	3,000	2,922	3,184	2,963	2,725	2,723	2,922	2,792
Crude protein (%)	23.00	0.00	6.82	6.82	20.00	20.00	19.60	19.60
Crude fat (%)	6.68	2.66	3.51	2.45	2.96	5.63	3.63	3.00
Calcium (%)	0.96	0.87	0.87	0.87	0.87	3.44	0.87	0.87
Available phosphorus (%)	0.48	0.43	0.44	0.43	0.44	1.56	0.44	0.44
Sodium (%)	0.16	0.16	0.16	0.16	0.16	0.23	0.16	0.16
Potassium (%)	1.00	0.41	0.90	0.90	1.00	1.00	0.90	0.90
Magnesium (%)	0.16	0.05	0.15	0.11	0.15	0.05	0.18	0.16
Chloride (%)	0.23	0.16	0.16	0.16	0.16	0.23	0.16	0.16

1 Vitamin mineral premix provided per kilogram of premix: vitamin A, 880,000 IU; vitamin D3, 330,000 IU; vitamin E, 4,000 IU; vitamin B12, 1,200 mcg; biotin, 22,000 mcg; menadione, 330 mg; thiamine, 400 mg; riboflavin, 800 mg; pantothenic acid, 1,500 mg; pyridoxine, 300 mg; niacin, 5,000 mg; folic acid, 100 mg; choline, 60,000 mg; iron, 6,000 mg; copper, 1,000 mg.

2 Nitrogen-free diet.

3 Soybean meal.

4 Pork meal.

5 Corn-soybean meal-pork meal.

6 Wheat-soybean meal-pork meal.

### Experimental Procedures and Sampling

On d 0, birds were weighed and randomly distributed into 84 cages (10 birds per cage). All birds had free access to water and the same basal starter diet until d 8. On d 9, birds were reweighed by cage and allocated in a completely randomized design to one of the 7 diets to give 12 replicates per diet based on cage BW. On day ten, 420 birds (6 replicates per diet on the left rows of cages) were orally gavaged with 1 mL of an *Eimeria* culture (100,000 oocysts of *E. acervulina* and 25,000 oocysts of *E. maxima*) in a distilled water suspension as described in Kim et al., 2017; Akbari Moghaddam Kakhki et al., 2019; Leung et al., 2019, while the other 6 replicates (non-challenged control on the right rows of cages) were given equal volumes of 0.9% saline in distilled water. The separation of right and left cages was an effort to minimize cross contamination of nonchallenged birds. The *Eimeria* culture (sourced from a local farm field strain in Hagersville, Ontario, Canada) and challenge protocols were provided by Dr. John Barta of the Department of Pathobiology, University of Guelph. Morbidity was not assessed prior to sample collection. On d 11, collection trays were inserted under the cages and excreta samples were collected from d 12 to 14 and stored at −20°C until required for analyses. On d 15, two birds per cage were bled via wing vein puncture; samples were collected into blood collection tubes with sodium heparin, placed on ice, and transported to the laboratory. The birds were subsequently euthanized and necropsied for lesion scores, to confirm infection, along the length of the intestine, as described by Price et al. (2014). Briefly, intestinal regions (duodenum, jejunum, and ileum) were assess blindly using a scale of 0 (none) to 4 (high) (Johnson and Reid, 1970). All the remaining birds were then euthanized by cervical dislocation and necropsied for ileal digesta samples. The ileum was located by excising 2 cm posterior to Meckel's diverticulum and 1 cm anterior to the ileocecal junction. The ileal digesta samples was pooled by cage, placed on ice, and transported back to the laboratory and stored at −20°C until required for analyses.

### Sample Processing and Laboratory Analysis

Blood samples were centrifuged at 2,500 × *g* for 15 min at 4°C and plasma was kept at −80°C. The excreta samples were thawed and pooled by cage. Both ileal digesta and excreta samples were subsequently freeze-dried and, along with samples of the test diets and test ingredients, finely ground for chemical analyses. Test diets and ingredients were analyzed for dry matter, gross energy, and nitrogen, while test diets, ingredients, and ileal digesta samples were analyzed for AA. Diet and ingredient samples were sent to METEX NØØVISTAGO (Amiens, France) laboratories for CP and AA analysis. Dry matter determination was carried out according to standard procedures (AOAC Intenational, 2005; method 930.15). Titanium content in diets and digesta was measured on a UV spectrophotometer following the method of Myers et al. (2004). Gross energy was determined in a bomb calorimeter (IKA Calorimeter System C 5000; IKA Works, Wilmington, NC). Nitrogen was determined by the combustion method (AOAC International, 2005; method 968.06) using a CNS-2000 carbon, N, and sulfur analyzer (LECO corporation, St. Joseph, MI). The crude protein values were then derived by multiplying a factor of 6.25 to the assayed N values. For AA analyses, digesta samples were prepared by acid hydrolysis according to AOAC (2005, method 982.30). Briefly, 100 mg of each sample was digested in 2.5 mL of concentrated HCl for 24 h at 110°C, followed by neutralization with 6N NaOH and cooled to room temperature. Feed and digesta samples for the analysis of sulfur-containing AA (Met and Cys) were subjected to performic acid oxidation prior to acid hydrolysis. Tryptophan was not determined. Plasma samples were prepared with 10% sulfosalicyclic acid for protein precipitation. The deproteinized plasma samples and neutralized digesta samples were then mixed with sodium borate buffer and analyzed using Ultra performance liquid chromatography (UPLC, Waters Corporation, Milford, CA).

### Calculations and Statistical Analysis

The apparent ileal digestibility (**AID**) and SID of AA were calculated according to Adeola et al. (2016).

AID, % = [1-(M_diet_/M_ileal_) x (AA_ileal_/AA_diet_)] × 100

Where M_diet_ and M_ileal_ represent the concentrations of the index marker (g/kg, DM) in the diet and ileal digesta, respectively, while AA_ileal_ and AA_diet_ represent AA concentrations (g/kg, DM) in the diet and ileal digesta, respectively.

Ileal total endogenous flow (**ITEF**) of AA was calculated according to Adedokun et al. (2007a):

ITEF of AA flow, mg/kg of DM intake = (AA_ileal_, mg/g) × [(M_diet,_ mg/g)/(M_ileal_ mg/g) × 1,000].

The equation for basal endogenous (BEL) of AA loss using NFD was:

BEL, mg/kg of DM intake = AA_ileal_ × (M_diet_/M_ileal_).

The equation for SID was:

SID, % = AID + (basal EAAL/ AA_diet_) × 100.

The data was subjected to a 6 × 2 factorial arrangement using mixed procedures of SAS version 9.4 (SAS Institute Inc, Cary, NC). The model included diet, challenge, and associated interaction as fixed factors. The data for BEL was analyzed using a one-way ANOVA with challenge as a fixed effect. An alpha level of 0.05 was used as the criterion for statistical significance.

## RESULTS

The analyzed chemical composition of feed ingredient samples is shown in Table 2. The analyzed CP and GE concentrations of the diets, shown in Table 3, were comparable for SBM and PM diets (20.6 vs. 19.2% and 3,958 vs. 3,839 kcal/kg, respectively), and for CSP and WSP diets (20.3 vs. 20.8% and 3,955 vs. 3,880 kcal/kg, respectively).

**Table 2:** Analyzed chemical composition of ingredients, as fed basis.

Item	Corn	Wheat	SBM^1^	PM^2^
Dry matter (%)	89.83	89.65	92.50	92.36
Gross energy (kcal/kg)	3,771	3,878	4,320	4,811
Crude protein (%)	7.94	12.40	49.90	52.40
Amino acids (%)				
Indispensable				
Arg	0.34	0.56	3.57	14.56
His	0.21	0.27	1.28	1.34
Ile	0.28	0.40	2.27	1.83
Leu	0.98	0.80	3.79	3.64
Lys	0.24	0.34	3.09	2.89
Met	0.14	0.19	0.65	0.47
Phe	0.39	0.54	2.52	2.09
Thr	0.28	0.36	1.94	5.18
Val	0.37	0.52	2.34	2.45
Dispensable				
Ala	0.59	0.45	2.13	0.10
Asp	0.53	0.65	5.59	0.90
Cys	0.15	0.28	0.69	0.30
Glu	1.45	3.34	8.97	2.93
Gly	0.29	0.51	2.06	2.53
Pro	0.69	1.14	2.53	4.33
Ser	0.38	0.55	2.51	2.28
Tyr	0.27	0.34	1.87	0.06

1 Soybean meal.

2 Pork meal.

**Table 3:** Analyzed chemical composition of experimental diets, as fed basis.

Item	NFD^1^	Corn	Wheat	SBM^2^	PM^3^	CSP^4^	WSP^5^
Dry matter (%)	89.77	89.66	89.36	90.24	90.00	90.17	89.84
Gross energy (kcal/kg)	3,612	3,751	3,683	3,958	3,839	3,955	3,880
Crude protein (%)	0.77	6.70	8.70	20.60	19.22	20.30	20.80
							
Amino acids (%)							
Indispensable							
Arg	0.04	0.28	0.41	1.46	0.46	1.31	1.32
His	0.02	0.17	0.19	0.53	0.51	0.50	0.49
Ile	0.03	0.23	0.29	0.95	0.78	0.85	0.86
Leu	0.08	0.79	0.57	1.62	1.45	1.68	1.50
Lys	0.03	0.19	0.27	1.28	1.19	1.14	1.10
Met	0.02	0.11	0.13	0.27	0.34	0.30	0.29
Phe	0.05	0.32	0.38	1.06	0.83	0.98	0.98
Thr	0.03	0.23	0.26	0.82	1.97	0.77	0.75
Val	0.05	0.30	0.37	0.99	0.99	0.94	0.95
Dispensable							
Ala	0.05	0.48	0.33	0.92	0.66	1.07	0.91
Asp	0.06	0.44	0.52	2.32	0.46	2.01	1.97
Cys	0.02	0.12	0.18	0.29	0.05	0.28	0.31
Glu	0.02	1.20	2.13	3.79	1.00	3.51	3.92
Gly	0.04	0.25	0.35	0.87	1.04	1.04	0.98
Pro	0.08	0.55	0.72	1.08	1.59	1.22	1.23
Ser	0.04	0.31	0.39	1.06	0.89	0.99	0.98
Tyr	0.02	0.21	0.23	0.74	0.38	0.70	0.68

1 Nitrogen-free diet.

2 Soybean meal.

3 Pork meal.

4 Corn-soybean meal-pork meal.

5 Wheat-soybean meal-pork meal.

Only *Eimeria*-challenged birds presented intestinal lesion scores higher than 0 (Table 4). There was a diet and challenge interaction (*P* < 0.05) on duodenum and jejunum lesion scores such that the challenged birds fed the mixed diets exhibited a higher severity of lesions compared to the birds fed NFD and birds fed NFD and corn diets, respectively. For the ileum, there was no (*P* > 0.05) diet and challenge interaction or diet effect on lesion scores. However, challenged birds had higher (*P* < 0.05) ileum lesion scores than nonchallenge birds. Challenge had no effect (*P* > 0.05) on plasma concentration of indispensable AA in birds fed NFD (Supplementary Table 1). There was an interaction effect (*P* <0.05) seen in plasma AA concentration such that Met concentration significantly increased with challenge for the mixed diets, while Arg, Gly, Pro, and Ser concentration significantly decreased with challenge in the PM diets. There was a challenge effect (*P* < 0.05) on plasma AA such that concentration for His, Thr, Asp, Gln, and Tyr decreased and that of Lys, Ile, Leu, and Val increased (Tables 5 and 6).

**Table 4:** Intestinal lesion scores in broiler chickens fed different feedstuff-based diets with or without *Eimeria* challenge.

Item		Duodenum	Jejunum	Ileum
Interaction effects				
Diet	*Eimeria*^1^			
NFD^2^	-	0.00^c^	0.00^d^	0.00
Corn	-	0.00^c^	0.00^d^	0.00
Wheat	-	0.00^c^	0.00^d^	0.00
SBM^3^	-	0.00^c^	0.00^d^	0.00
PM^4^	-	0.00^c^	0.00^d^	0.00
CSP^5^	-	0.00^c^	0.00^d^	0.00
WSP^6^	-	0.00^c^	0.00^d^	0.00
NFD	+	2.00^b^	1.25^c^	0.25
Corn	+	2.75^a^	1.42^c^	0.58
Wheat	+	3.08^a^	2.17^ab^	0.83
SBM	+	3.00^a^	2.17^ab^	0.83
PM	+	2.83^a^	1.75^bc^	0.50
CSP	+	3.25^a^	2.58^a^	1.00
WSP	+	3.00^a^	2.42^ab^	0.67
SEM		0.40	0.29	0.10
Main effects of the diet				
	NFD	1.00	0.63	0.13
	Corn	1.38	1.25	0.42
	Wheat	1.54	0.31	0.15
	SBM	1.50	1.09	0.42
	PM	1.42	0.88	0.25
	CSP	1.63	1.29	0.50
	WSP	1.50	1.21	0.34
SEM		0.08	0.14	0.05
Main effects of Eimeria				
-		0.00	0.00	0.00^b^
+		2.84	1.97	0.67^a^
SEM		0.40	0.29	0.10
Probabilities				
Diet		0.002	<0.001	0.082
*Eimeria*		<0.001	<0.001	<0.001
Diet x *Eimeria*		0.002	<0.001	0.082

1 “-” unchallenged, “+” challenged.

2 Nitrogen-free diet.

3 Soybean meal.

4 Pork meal.

5 Corn-soybean meal-pork meal.

6 Wheat-soybean meal-pork meal. N=6. ^a-d^Means assigned different letters within a response criterion are significantly different, *P* < 0.05.

**Table 5:** Plasma concentration (*μ*M) of indispensable amino acids in broiler chickens fed different feedstuff-based diets with or without *Eimeria* challenge.

Item		Arg	His	Ile	Leu	Lys	Met	Phe	Thr	Trp	Val	Mean
Interaction effects												
Diet	*Eimeria*^1^											
Corn	-	179.50^d^	175.50	57.17	173.67	101.67	53.83^abc^	125.67	252.67	48.83	150.67	132.00
Wheat	-	214.50^cd^	152.17	57.83	111.50	46.00	34.17^bcd^	102.83	206.33	57.50	157.33	114.00
SBM^2^	-	396.50^bc^	192.33	152.83	220.83	938.67	22.33^d^	182.33	1,281.67	103.67	339.33	383.17
PM^3^	-	713.33^a^	109.33	95.50	176.00	459.33	70.00^a^	126.33	801.33	53.00	300.83	290.67
CSP^4^	-	476.17^b^	177.83	116.67	239.33	526.50	20.00^d^	164.50	844.33	88.67	260.67	291.33
WSP^5^	-	500.67^b^	199.17	119.50	180.83	409.33	20.50^d^	155.50	960.67	106.17	270.17	292.17
Corn	+	168.83^d^	113.50	61.33	184.17	114.67	48.17^abcd^	127.83	194.33	46.67	161.67	122.17
Wheat	+	273.33^cd^	95.00	70.83	144.67	60.50	35.00^bcd^	111.33	174.83	57.00	188.83	121.17
SBM	+	332.00^bcd^	125.50	127.67	196.67	831.83	29.33^cd^	157.67	936.17	99.50	295.00	313.17
PM	+	360.33^bcd^	71.67	94.17	179.83	675.00	67.67^a^	110.67	624.17	49.17	272.17	250.50
CSP	+	318.33^bcd^	113.50	126.33	232.50	1002.17	52.17^abc^	151.00	790.50	76.17	288.50	315.00
WSP	+	346.00^bcd^	99.33	128.33	209.50	783.67	62.17^ab^	134.83	698.67	67.50	278.00	280.50
SEM		44.37	12.23	9.42	10.46	102.34	5.31	7.08	104.77	6.59	18.62	27.06
Main effects of diet												
	Corn	174.17	144.50^a^	59.25^d^	178.92^abc^	108.17^c^	51.00	126.75^bc^	223.50^c^	47.75^b^	156.17^b^	127.09^c^
	Wheat	243.92	123.59^ab^	64.33^cd^	128.09^c^	53.25^c^	34.59	107.08^c^	190.58^c^	57.25^b^	173.08^b^	117.59^c^
	SBM	364.25	158.92^a^	140.25^a^	208.75^ab^	885.25^a^	25.83	170.00^a^	1108.92^a^	101.59^a^	317.17^a^	348.17^a^
	PM	536.83	90.50^b^	94.84^bc^	177.92^bc^	567.17^b^	68.84	118.50^c^	712.75^b^	51.09^b^	286.50^a^	270.59^b^
	CSP	397.25	145.67^a^	121.50^ab^	235.92^a^	764.34^ab^	36.09	157.75^ab^	817.42^ab^	82.42^a^	274.59^a^	303.17^ab^
	WSP	423.34	149.25^a^	123.92^ab^	195.17^ab^	596.50^ab^	41.34	145.17^abc^	829.67^ab^	86.84^a^	274.09^a^	286.34^ab^
SEM		53.11	10.15	13.68	14.78	139.66	6.19	9.88	149.19	9.02	26.89	39.36
Main effects of *Eimeria*												
-		413.45	167.72^a^	99.92	183.69	413.58^b^	36.81	142.86	724.50^a^	76.31^a^	246.50	250.56
+		299.80	103.08^b^	101.44	191.22	577.97^a^	49.09	132.22	569.78^b^	66.00^b^	247.36	233.75
SEM		56.82	32.32	0.76	3.77	82.20	6.14	5.32	77.36	5.15	0.43	8.40
Probabilities												
Diet		<0.001	0.003	<0.001	<0.001	<0.001	<0.001	<0.001	<0.001	<0.001	<0.001	<0.001
*Eimeria*		0.001	<0.001	0.824	0.509	0.009	0.001	0.165	0.010	0.038	0.953	0.266
Diet × *Eimeria*		0.002	0.490	0.629	0.695	0.055	0.001	0.774	0.534	0.209	0.581	0.524

N = 6.

1 “-” unchallenged, “+” challenged

2 Soybean meal.

3 Pork meal.

4 Corn-soybean meal-pork meal.

5 Wheat-soybean meal-pork meal. ^a-d^Means assigned different letters within a response criterion are significantly different, *P* < 0.05.

**Table 6:** Plasma concentration (*μ*M) of dispensable amino acids in broiler chickens fed different feedstuff-based diets with or without *Eimeria* challenge.

Item		Ala	Asp	Cys	Glu	Gly	Pro	Ser	Tyr	Mean
Interaction effects										
Diet	*Eimeria*^1^									
Corn	-	710.00	56.00	70.80	125.30	832.20^bc^	332.00^d^	1,094.50^bc^	130.00	418.8^b^
Wheat	-	497.30	52.50	71.00	142.80	681.30^bc^	370.80^d^	653.80^c^	138.30	326.0^b^
SBM^2^	-	772.50	128.00	119.80	158.80	710.20^c^	505.50^bcd^	837.30^bc^	141.80	421.8^b^
PM^3^	-	951.70	99.70	76.20	182.20	2,924.80^a^	845.50^a^	1,913.20^a^	86.80	885.2^a^
CSP^4^	-	888.30	70.00	83.20	156.50	944.80^bc^	634.20^ab^	880.70^b^	154.00	476.5^b^
WSP^5^	-	649.30	85.30	120.30	177.50	755.30^bc^	607.00^bc^	786.70^bc^	130.50	414.2^b^
Corn	+	646.50	39.70	58.80	114.00	784.70^bc^	334.20^d^	965.80^bc^	127.50	384.0^b^
Wheat	+	526.80	49.50	87.50	143.00	585.80^c^	404.30^cd^	791.20^bc^	138.20	340.7^b^
SBM	+	702.00	95.00	101.70	153.30	763.70^bc^	469.00^bcd^	890.50^bc^	137.50	414.0^b^
PM	+	776.00	56.30	48.50	129.80	1,246.50^b^	504.30^bcd^	1,250.20^b^	66.70	509.7^b^
CSP	+	704.70	65.80	98.80	171.50	763.20^bc^	469.80^bcd^	959.80^bc^	137.00	421.2^b^
WSP	+	863.30	80.00	89.70	156.50	704.20^c^	522.70^bcd^	950.70^bc^	99.50	433.2^b^
SEM		39.53	7.35	6.42	6.05	183.65	42.00	94.24	7.45	41.80
Main effects of diet										
	Corn	678.25^ab^	47.85^b^	64.80^c^	119.65	808.45	333.10	1,030.15	128.75^a^	401.40
	Wheat	512.05^b^	51.00^b^	79.25^bc^	142.90	633.55	387.55	722.50	138.25^a^	333.35
	SBM	737.25^ab^	111.50^a^	110.75^a^	156.05	736.95	487.25	863.90	139.65^a^	417.90
	PM	863.85^a^	78.00^ab^	62.35^c^	156.00	2,085.65	674.90	1,581.70	76.75^b^	697.45
	CSP	796.50^a^	67.90^ab^	91.00^abc^	164.00	854.00	552.00	920.25	145.50^a^	448.85
	WSP	756.30^ab^	82.65^ab^	105.00^ab^	167.00	729.75	564.85	868.70	115.00^ab^	423.70
SEM		49.35	9.56	8.27	7.13	224.30	51.04	123.61	10.40	51.26
Main effects of *Eimeria*										
-		744.85	81.92	90.22	157.18	1,141.43	549.17	1,027.70	130.23	490.42
+		703.22	64.38	80.83	144.68	808.02	450.72	968.03	117.73	417.13
SEM		20.82	8.77	4.69	6.25	166.71	49.23	29.83	6.25	36.64
Probabilities										
Diet		0.002	0.004	<0.001	0.087	<0.001	<0.001	<0.001	0.001	<0.001
*Eimeria*		0.391	0.073	0.121	0.216	<0.001	0.001	0.396	0.164	0.003
Diet × *Eimeria*		0.180	0.766	0.087	0.503	<0.001	0.002	0.010	0.904	<0.001

N = 6.

1 “-” unchallenged, “+” challenged.

2 Soybean meal.

3 Pork meal.

4 Corn-soybean meal-pork meal.

5 Wheat-soybean meal-pork meal. ^a-d^Means assigned different letters within a response criterion are significantly different, *P* < 0.05.

The ileal total flow of endogenous and undigested (**ITEF**) AA is shown in Tables 7 and 8. An interaction effect (*P* < 0.05) on ITEF was seen for all indispensable AA except for Arg and Met (Table 7) and for most dispensable AA except for Ala, Asp, and Cys (Table 8). Specifically, challenge increased (*P* < 0.05) ITEF of Lys, Thr, and mean of indispensable and dispensable AA in protein feedstuffs (SBM and PM) and mixture of ingredients but not in cereal grains. In general, the main effect of challenge increased total ITEF (*P* < 0.05) for all AA except for Met. The values for the BEL of AA are shown in Table 9. While there was no challenge effect (*P* > 0.05) seen in the BEL of AA, however, these values were numerically higher in challenged birds.

**Table 7:** Ileal total endogenous flow (mg/kg DM) of indispensable amino acids in broiler chickens fed different feedstuff-based diets with or without *Eimeria* challenge.

Item		Arg	His	Ile	Leu	Lys	Met	Phe	Thr	Val	Mean
Interaction effects											
Diet	*Eimeria*^1^										
Corn	-	1,134.14	751.76^d^	1,153.52^de^	2,076.28^c^	836.56^d^	233.05	1,100.83^d^	926.52^d^	1,552.32^e^	1,085.00^d^
Wheat	-	524.85	575.71^d^	920.52^e^	1507.23^c^	1,140.26^cd^	374.02	888.44^d^	1,367.05^d^	1,418.61^e^	968.52^d^
SBM^2^	-	1,987.00	1,144.12^cd^	1,866.58^bcde^	3,195.73^bc^	1,908.73^bcd^	371.65	1,949.35^bcd^	2,282.24^cd^	2,224.93^de^	1,881.15^cd^
PM^3^	-	2,617.61	1,109.48^cd^	1,656.45^de^	3,110.48^bc^	2,394.61^bc^	385.41	1,745.19^cd^	2,272.96^cd^	2,527.45^cde^	1,979.96^cd^
CSP^4^	-	1,834.77	864.27^cd^	1,371.26^de^	2,431.21^c^	1,553.97^cd^	220.24	1,399.10^d^	1,680.79^cd^	1,713.79^de^	1,452.16^cd^
WSP^5^	-	2,870.93	834.58^d^	1,435.79^de^	2,463.35^c^	1,721.82^cd^	457.94	1,418.03^d^	1,801.34^cd^	1,849.81^de^	1,650.40^cd^
Corn	+	1,502.77	873.97^cd^	1,218.81^de^	2,193.85^c^	1,058.83^d^	396.09	1,175.47^d^	1,503.22^cd^	1,657.28^de^	1,286.70^d^
Wheat	+	1,332.04	1,261.36^cd^	1,777.71^cde^	3024.95^bc^	1,685.36^cd^	486.43	1,736.06^d^	2,461.20^cd^	2,544.45^cde^	1,812.17^cd^
SBM	+	3,549.87	2,213.84^ab^	3,164.82^ab^	5,464.36^a^	3,731.06^a^	625.89	3,341.56^a^	4,101.68^ab^	4,090.89^abc^	3,364.89^ab^
PM	+	3,964.98	2,588.57^a^	3,083.02^abc^	5,443.75^a^	4,327.73^a^	336.40	3,074.48^ab^	4,085.90^ab^	4,212.53^ab^	3,457.48^ab^
CSP	+	3,774.16	1,694.23^bc^	2,476.93^abcd^	4,750.10^ab^	3,067.57^ab^	240.72	2,750.73^abc^	3,057.33^bc^	3,243.16^bcd^	2,783.88^bc^
WSP	+	4,894.65	2,291.18^ab^	3,744.26^a^	5,723.06^a^	4,138.23^a^	604.93	3,363.48^a^	4,966.56^a^	5,009.13^a^	3,859.50^a^
SEM		385.23	196.14	263.85	430.83	354.47	38.41	260.21	363.76	346.63	285.15
Main effects of diet											
	Corn	1,318.45^c^	812.80	1,186.15	2,135.10	947.70	314.60	1,138.15	1,214.85	1,604.80	1,185.85
	Wheat	928.40^c^	918.55	1,349.10	2,266.05	1,412.85	430.20	1,312.25	1,914.15	1,981.55	1,390.35
	SBM	2,768.45^b^	1,678.95	2,515.70	4,330.05	2,819.90	498.75	2,645.50	3,191.95	3,157.90	2,623.05
	PM	3,291.25^ab^	1,849.05	2,369.70	4,277.10	3,361.15	360.90	2,409.85	3,179.45	3,370.00	2,718.75
	CSP	2,804.50^b^	1,279.25	1,924.10	3,590.65	2,310.75	230.45	2,074.90	2,369.05	2,478.50	2,118.05
	WSP	3,882.75^a^	1,562.90	2,590.05	4,093.25	2,930.00	531.40	2,390.75	3,383.95	3,429.45	2,754.95
SEM		467.93	171.50	247.78	409.18	383.36	46.66	255.51	352.28	313.59	283.95
Main effects of *Eimeria*											
-		1,828.20^b^	879.98	1,400.68	2,464.05	1,592.65	340.37	1,416.82	1,721.82	1,881.15	1,502.88
+		3,169.73^a^	1,820.52	2,577.58	4,433.35	3,001.47	448.40	2,573.65	3,362.65	3,459.58	2,760.78
SEM		670.77	470.27	588.45	984.65	704.41	54.02	578.42	820.42	789.22	628.95
Probabilities											
Diet		<0.001	<0.001	<0.001	<0.001	<0.001	0.194	<0.001	<0.001	<0.001	<0.001
*Eimeria*		<0.001	<0.001	<0.001	<0.001	<0.001	0.155	<0.001	<0.001	<0.001	<0.001
Diet × *Eimeria*		0.159	0.003	0.008	0.017	0.001	0.881	0.011	0.007	0.004	0.003

N = 6.

1 “-” unchallenged, “+” challenged.

2 Soybean meal.

3 Pork meal.

4 Corn-soybean meal-pork meal.

5 Wheat-soybean meal-pork meal. ^a-e^Means assigned different letters within a response criterion are significantly different, *P* < 0.05.

**Table 8:** Ileal total endogenous flow (mg/kg) of dispensable amino acids in broiler chickens fed different feedstuff-based diets with or without *Eimeria* challenge.

Item		Ala	Asp	Cys	Glu	Gly	Pro	Ser	Tyr	Mean
Interaction effects										
Diet	*Eimeria*^1^									
Corn	-	1,199.85	414.71	95.00	2,500.82^ef^	766.96^de^	1,292.22^de^	1,522.86^cd^	479.26^bc^	1,033.96^de^
Wheat	-	375.61	374.94	183.56	2,045.70^f^	381.12^e^	1,178.47^e^	1,207.29^d^	282.61^bc^	753.66^e^
SBM^2^	-	458.78	152.42	108.48	3,808.35^def^	2,519.40^bcd^	1,782.50^de^	2,389.44^bcd^	535.41^bc^	1,469.35^cde^
PM^3^	-	2,681.94	557.87	84.37	5,650.09^bcde^	4,341.40^b^	3,008.07^bcd^	2,242.05^bcd^	166.76^c^	2,341.57^bcd^
CSP^4^	-	269.84	568.48	86.58	3,591.35^ef^	2,446.61^cd^	2,067.24^cde^	1,889.07^cd^	814.10^ab^	1,466.66^cde^
WSP^5^	-	150.38	560.42	90.22	3,344.44^ef^	2,853.75^bc^	1,788.79^de^	1,944.76^cd^	609.26^bc^	1,417.75^cde^
Corn	+	1,597.81	504.43	202.40	2,799.66^ef^	923.88^de^	2,046.36^de^	1,848.12^cd^	514.97^bc^	1,304.70^de^
Wheat	+	679.78	412.84	650.33	4,470.20^cdef^	379.81^e^	2,618.47^bcde^	2,570.04^d^	345.89^bc^	1,515.92^cde^
SBM	+	594.46	261.23	257.27	7,597.09^abc^	4,180.27^bc^	4,405.98^ab^	4,167.35^ab^	796.35^ab^	2,782.50^ab^
PM	+	3,116.16	926.98	167.27	8,292.23^ab^	8,514.99^a^	5,488.04^a^	4,998.79^a^	194.88^bc^	3,962.42^a^
CSP	+	348.45	747.76	140.13	6,817.72^abcd^	4,266.23^bc^	3,857.20^abc^	3,300.19^abc^	1,358.43^a^	2,604.51^bc^
WSP	+	176.03	725.56	203.38	9,871.60^a^	3,129.84^bc^	4,951.13^a^	5,012.17^a^	1,317.72^a^	3,173.43^ab^
SEM		288.71	62.23	45.00	734.60	666.67	423.07	379.34	113.95	281.08
Main effects of diet										
	Corn	1, 398.80^b^	459.55^bc^	148.70	2,650.25	845.40	1,669.30	1,685.45	497.10	1,169.30
	Wheat	527.70^c^	393.85^cd^	416.95	3,257.95	380.45	1,898.50	1,888.65	314.25	1,134.80
	SBM	526.65^c^	206.80^d^	182.90	5,702.75	3,349.85	3,094.25	3,278.40	665.87	2,125.95
	PM	2,899.05^a^	742.45^a^	125.85	6,971.15	6,428.20	4,248.05	3,620.40	180.85	3,152.00
	CSP	309.15^c^	658.15^ab^	113.35	5,204.55	3,356.40	2,962.20	2,594.65	1,086.25	2,035.60
	WSP	163.20^c^	643.00^ab^	146.80	6,608.00	2,991.80	3,369.95	3,478.50	963.50	2,295.60
SEM		423.67	81.92	46.59	719.65	883.86	391.29	339.93	145.93	309.25
Main effects of *Eimeria*										
-		856.05^b^	438.13^b^	108.05^b^	3,490.13	2,218.20	1,852.88	1,865.90	481.25	1,413.85
+		1,085.47^a^	596.47^a^	270.13^a^	6,641.42	3,565.83	3,894.53	3,649.45	754.69	2,557.23
SEM		114.71	79.17	81.04	1,575.64	673.82	1,020.83	891.78	136.72	571.69
Probabilities										
Diet		<0.001	<0.001	0.221	<0.001	<0.001	<0.001	<0.001	<0.001	<0.001
*Eimeria*		0.014	0.003	0.042	<0.001	<0.001	<0.001	<0.001	0.001	<0.001
Diet × *Eimeria*		0.696	0.273	0.664	0.001	<0.001	0.029	0.020	0.042	0.020

N = 6.

1 “-” unchallenged, “+” challenged.

2 Soybean meal.

3 Pork meal.

4 Corn-soybean meal-pork meal.

5 Wheat-soybean meal-pork meal. ^a-f^Means assigned different letters within a response criterion are significantly different, *P* < 0.05.

**Table 9:** Basal endogenous amino acids loss (mg/kg DMI) in broiler chickens fed nitrogen free diet with or without *Eimeria* challenge^1^.

Item	BEL -	BEL +	SEM	Probabilities
Indispensable				
Arg	1,050	1,560	250	0.369
His	270	340	30	0.556
Ile	340	610	140	0.189
Leu	510	840	170	0.345
Lys	250	490	120	0.465
Met	340	250	50	0.622
Phe	350	500	80	0.452
Thr	600	1,100	250	0.197
Val	690	950	130	0.465
Mean	490	740	120	0.268
Dispensable				
Ala	650	590	30	0.817
Asp	350	1,160	410	0.117
Cys	210	220	10	0.986
Glu	960	1,140	90	0.742
Gly	170	230	30	0.973
Pro	310	780	240	0.059
Ser	650	890	120	0.323
Tyr	220	400	90	0.464
Mean	440	680	120	0.291

N = 6.

1 “-” unchallenged, “+” challenged.

The SID values (Tables 10 and 11) were calculated by correcting AID values (Supplementary Tables 2 and 3) for BEL of AA. There was an interaction (*P* < 0.05) between diet and challenge on SID of Arg, Thr, and Val; such that *Eimeria* decreased SID of Arg in birds fed wheat diet, SID of Thr in birds fed corn and WSP diets and SID of Val in birds fed SBM and WSP diets. For dispensable AA, interaction (*P* < 0.05) between challenge and diet was such that, *Eimeria* decreased SID of Glu in PM and WSP diets and SID of Gly in birds fed PM diet. The effect of coccidial challenge was significant (*P* < 0.05) for the SID of most AA with exception to Met, Asp, and Cys, such that the SID values decreased in the challenged birds compared to the unchallenged birds. The largest impact was seen on Lys, His, Ser, Thr, and Pro with the magnitude of difference of 45.8, 34.5, 33.5, 32.9%, and 27.2%, respectively. With exception of Arg, Thr, Asp, and Cys, birds fed mixture had higher (*P* < 0.05) SID values compared to birds fed single ingredients.

**Table 10:** Standardized ileal digestibility (%) of indispensable amino acids in broiler chickens fed different feedstuff-based diets with or without *Eimeria* challenge.

Item		Arg	His	Ile	Leu	Lys	Met	Phe	Thr	Val	Mean
Interaction effects											
Diet	*Eimeria*^1^										
Corn	-	92.55^ab^	59.63	48.26	72.67	58.60	84.03	60.90	51.99^ef^	50.81^cde^	64.38
Wheat	-	97.09^a^	61.42	59.41	66.54	22.13	70.14	67.96	63.27^bcde^	52.03^cde^	62.22
SBM^2^	-	89.62^ab^	72.05	72.14	73.35	79.84	82.77	72.33	76.60^abcd^	69.63^abc^	76.48
PM^3^	-	80.40^abc^	79.15	77.31	80.13	80.64	89.83	80.03	90.39^a^	76.53^ab^	81.60
CSP^4^	-	91.51^ab^	82.15	81.02	84.98	85.24	92.88	83.03	78.58^abc^	81.01^a^	84.49
WSP^5^	-	89.10^ab^	80.49	79.27	80.62	82.20	88.70	79.81	72.89^abcd^	77.64^ab^	81.19
Corn	+	79.53^bc^	53.16	46.21	72.19	48.21	71.74	58.66	22.56^g^	48.24^de^	55.61
Wheat	+	66.50^c^	26.25	36.76	44.38	−14.70	60.58	48.99	43.88^ef^	36.90^e^	38.84
SBM	+	80.51^abc^	45.38	52.88	54.37	60.61	70.05	52.24	58.01^de^	43.24^de^	57.48
PM	+	70.67^c^	50.43	62.33	65.09	65.16	90.99	64.41	82.80^ab^	60.27^bcd^	68.02
CSP	+	79.98^bc^	64.20	65.72	70.05	70.88	91.72	65.80	61.09^cde^	62.59^abcd^	70.23
WSP	+	80.63^abc^	45.11	52.56	53.87	56.88	83.51	50.99	32.80^fg^	46.18^de^	55.84
SEM		2.62	4.89	4.13	3.50	8.35	3.08	3.38	5.96	4.26	3.88
Main effects of diet											
	Corn	86.04	56.40^bc^	47.24^b^	72.43^ab^	53.41^b^	77.89^ab^	59.78^c^	37.28	49.53	60.00^bc^
	Wheat	81.80	43.84^c^	48.09^b^	55.46^c^	3.72^c^	65.36^b^	58.48^c^	53.58	44.47	50.53^c^
	SBM	85.07	58.72^abc^	62.51^a^	63.86^bc^	70.23^ab^	76.41^ab^	62.29^bc^	67.31	56.44	66.98^ab^
	PM	75.54	64.79^ab^	69.82^a^	72.61^ab^	72.90^a^	90.41^a^	72.22^ab^	86.60	68.40	74.81^a^
	CSP	85.75	73.18^a^	73.37^a^	77.52^a^	78.06^a^	92.30^a^	74.42^a^	69.84	71.80	77.36^a^
	WSP	84.87	62.80^ab^	65.92^a^	67.25^abc^	69.54^ab^	86.11^a^	65.40^abc^	52.85	61.91	68.52^a^
SEM		1.65	4.00	4.52	3.19	11.37	4.15	2.69	6.97	4.35	4.04
Main effects of *Eimeria*											
-		90.05	72.48^a^	69.57^a^	76.38^a^	68.11^a^	84.73	74.01^a^	72.29	67.94	75.06^a^
+		76.30	47.42^b^	52.74^b^	59.99^b^	47.84^b^	78.10	56.85^b^	50.19	49.57	57.67^b^
SEM		6.87	12.53	8.41	8.20	10.13	3.31	8.58	11.05	9.19	8.69
Probabilities											
Diet		0.021	<0.001	<0.001	<0.001	<0.001	0.001	0.001	<0.001	<0.001	<0.001
*Eimeria*		<0.001	<0.001	<0.001	<0.001	<0.001	0.080	<0.001	<0.001	<0.001	<0.001
Diet × *Eimeria*		0.019	0.067	0.186	0.071	0.317	0.840	0.058	0.007	0.029	0.226

N = 6.

1 “-” unchallenged, “+” challenged.

2 Soybean meal.

3 Pork meal.

4 Corn-soybean meal-pork meal.

5 Wheat-soybean meal-pork meal. ^a-g^Means assigned different letters within a response criterion are significantly different, *P* < 0.05.

**Table 11:** Standardized ileal digestibility (%) of dispensable amino acids in broiler chickens fed different feedstuff-based diets with or without *Eimeria* challenge.

Item		Ala	Asp	Cys	Glu	Gly	Pro	Ser	Tyr	Mean
Interaction effects										
Diet	*Eimeria*^1^									
Corn	-	66.76	64.84	94.53	70.13^de^	74.22^abc^	70.66	56.39	56.81	69.29
Wheat	-	75.03	63.61	84.57	93.32^a^	80.14^a^	65.36	62.46	61.45	73.24
SBM^2^	-	78.97	93.25	97.59	90.97^ab^	75.37^abc^	81.37	69.96	78.25	83.22
PM^3^	-	38.13	88.94	88.50	49.82^f^	51.74^d^	82.02	76.86	56.83	66.61
CSP^4^	-	87.75	81.03	98.01	94.12^a^	76.83^ab^	84.70	80.80	77.48	85.09
WSP^5^	-	75.78	77.52	98.04	94.30^a^	70.54^abc^	85.70	76.57	85.66	83.01
Corn	+	54.76	59.77	87.53	66.76^e^	69.04^abcd^	54.56	47.42	54.13	61.75
Wheat	+	52.94	69.04	82.74	82.34^abcd^	80.40^a^	35.70	29.03	65.93	62.27
SBM	+	72.00	90.07	93.43	79.68^bcde^	58.99^cd^	54.03	46.90	67.85	70.37
PM	+	36.27	83.46	86.76	20.62^g^	18.69^e^	67.91	46.62	49.99	49.99
CSP	+	83.12	77.99	96.23	85.91^abc^	59.33^bcd^	72.33	65.42	62.53	75.36
WSP	+	71.06	73.23	94.70	77.52^cde^	65.77^abcd^	60.19	45.59	73.20	70.16
SEM		4.87	3.18	1.60	6.28	4.95	4.33	4.60	3.16	2.94
Main effects of diet										
	Corn	60.76^c^	62.31^e^	91.03^abc^	68.45	71.63	62.61^bc^	51.91^b^	55.47^bc^	65.52^bc^
	Wheat	63.99^bc^	66.33^de^	83.66^c^	87.83	80.27	50.53^c^	45.75^b^	63.69^abc^	67.76^b^
	SBM	75.49^ab^	91.66^a^	95.51^ab^	85.33	67.18	67.70^ab^	58.43^ab^	73.05^ab^	76.80^a^
	PM	37.20^d^	86.20^ab^	87.63^bc^	35.22	35.22	74.97^ab^	61.74^ab^	53.41^c^	58.30^c^
	CSP	85.44^a^	79.51^bc^	97.12^a^	90.02	68.08	78.52^a^	73.11^a^	70.01^abc^	80.23^a^
	WSP	73.42^ab^	75.38^cd^	96.37^a^	85.91	68.16	72.95^ab^	61.08^ab^	79.43^a^	76.59^a^
SEM		6.79	4.61	2.22	8.64	6.29	4.15	3.81	4.17	3.42
Main effects of *Eimeria*										
-		70.40^a^	78.20	93.54	82.11	71.47	78.30^a^	70.51^a^	69.41	76.74^a^
+		61.69^b^	75.59	90.23	68.81	58.70	57.45^b^	46.83^b^	62.27	64.98^b^
SEM		4.36	1.30	1.65	6.65	6.39	10.42	11.84	3.57	5.88
Probabilities										
Diet		<0.001	<0.001	<0.001	<0.001	<0.001	<0.001	0.001	0.001	<0.001
*Eimeria*		0.005	0.216	0.059	<0.001	<0.001	<0.001	<0.001	0.068	<0.001
Diet × *Eimeria*		0.161	0.704	0.941	0.001	0.001	0.192	0.219	0.728	0.512

N = 6.

1 “-” unchallenged, “+” challenged.

2 Soybean meal.

3 Pork meal.

4 Corn-soybean meal-pork meal.

5 Wheat-soybean meal-pork meal. ^a-g^Means assigned different letters within a response criterion are significantly different, *P* < 0.05.

## DISCUSSION

For the current study, we observed a diet and challenge interaction effect on duodenum and jejunum lesion scores, compared to the ileum where only a challenge effect was seen. There are seven species of *Eimeria* that have been recognized to be pathogenic along the intestinal tract (Chapman, 2014). In the current study, we used *E. acervulina* and *E. maxima*, species that typically afflicts the duodenum and jejunum, respectively. We observed more severe lesion scores in the challenged birds fed the mixed diets compared to the NFD and/or corn diet, suggesting that dietary AA profile may influence *Eimeria* pathogenicity. Previous research has shown that low levels of dietary protein can decrease the severity of coccidiosis as the reduction in trypsin activity in the small intestine limits sporozoite excystation and subsequent parasitic invasion (Britton et al., 1964; Ding et al., 2016; Jenkins et al., 2019). In this manner, it may indicate why decreased severity of lesion scores was observed these diet groups (NFD and corn) compared to the mixed diet groups. Although it is unclear as to why lesion severity was similar across many of the dietary treatments, irrespective of the dietary CP level, in the jejunum. Perhaps the difference seen in the NFD and corn compared to the mixed diets is apparent because while all the diets did not meet AA requirements, the mixed diets were theoretically approaching complete diet in the context of this experiment.

Plasma AA concentrations represent the net effect of AA appearance from tissue release and dietary absorption and AA disappearance for protein synthesis, oxidation, and metabolism (Cynober, 2002). Although results should be interpreted with caution due to the dynamic nature of this free AA pool, which can be influenced by a number of different factors including dietary intake, plasma AA concentrations can be useful in revealing low concentrations of AA as an indication of limiting AA (Fernández-Fígares et al., 2005). There was an interaction effect seen in plasma AA concentration such that Met concentration increased with challenge for the mixed diets, while Arg, Gly, Pro, and Ser concentrations decreased with challenge in the PM diets, suggesting that the *Eimeria* response was dependent on the diet type. As the mixed diets were formulated to be comparable to a standard commercial diet, it was interesting to see the increase in plasma Met concentration, indicating a greater supply than metabolic demand, during an *Eimeria* challenge compared to the decrease in plasma Arg, Gly, Pro, and Ser concentration in the PM diets where the imbalance of the aforementioned AA supply in the diet, as well as the changes in AA utilization due to the challenge, may have influenced the vulnerability to AA limitation.

Challenge decreased plasma concentrations of His, Thr, Asp, Gln, and Tyr and increased plasma concentrations of Lys, Ile, Leu, and Val. These results were generally in agreement with that of Rochell et al. (2016), where plasma concentrations of Arg and Gln decreased with increasing *E. acervulina* inoculation dose and concentrations of Lys, Leu, Ile, and Val increased with increasing *E. acervulina* dose. The reduced Arg concentration may have been related to the increased demand for Arg as a substrate in the biosynthesis of nitric oxide, an important mediator of innate and acquired immunity in response to *Eimeria* (Allen, 1999). Gln is used to produce energy for the GIT (Rao and Samak, 2012). Therefore, reduced plasma concentrations of Gln observed in challenged birds may have been due to the increased demand for energy to fuel the immune cells and enterocytes. Conversely, the increase in Lys and branched chain AA concentrations may have been related to the reduced muscle protein synthesis perhaps induced by imbalanced AA availability.

Endogenous losses represent the nondietary AA synthesized for metabolic function, such as enzymes or sloughed cells, that have not been absorbed at the end of the small intestine (Adeola et al., 2016). Even though basal endogenous losses represent the quantity of AA that are lost from the animal regardless of the ingredient composition, these losses can be influenced by dry matter intake and/or the animal's physiological state (Stein et al., 2007). Although we did not measure feed intake in this study, pathogenesis of *Eimeria* entails invasion of the intestinal cells as part of the life cycle resulting in intestinal damage and thus increased mucogenesis. However, although *Eimeria* induced epithelial cell damage and as indicated by lesion scores, BEL of AA was not affected. Teng et al. (2021) observed linear and quadratic increases in BEL in responses to *E. maxima* challenge at 0, 15,000, 25,000, and 50,000 oocysts. However, a contrast that compared nonchallenged control vs. average of all challenged groups indicated that there were no statistical differences on BEL (Teng et al., 2021). Indeed, the BEL of some AA in 15,000 and 25,000 oocysts groups were similar or numerically less than the control group. Adedokun et al. (2016) also observed that coccidiosis vaccine challenge reduced endogenous loss of AA. Thus, taking aforementioned studies and plasma AA concentration and BEL data in the current study into considerations, it appears that *Eimeria* challenge does not influence basal endogenous losses of AA from sloughed epithelial cells, enzymes, and mucin. Coccidia infection strip off the mucosa as evidenced by disrupted intestinal histomorphology (Kim et al., 2017; Leung et al, 2019; Lu et al., 2019). Adedokun et al. (2016) opined that the reduced intestinal mucosa area in coccidia afflicted birds minimized endogenous losses of AA. Thus, the observed ITEF of AA in the current study was mainly due to specific endogenous losses and undigested AA representing *Eimeria* induced increase in undigested AA at terminal ileum.

Whereas challenge did not affect SID of Met, there was a diet effect such that SID of Met of the wheat diet was significantly lower than that of the PM and mixed diets. In addition, the SID of Met of the corn and SBM diets were numerically lower than that of the PM and mixed diets. Methionine is typically the first limiting AA in broilers fed grain, such as corn and wheat, diets and is therefore, supplemented to ensure optimal protein metabolism and immune function (Lai et al., 2018). In the current study, diets were not supplemented with additional crystalline AA to meet ideal AA ratios, but rather formulated on a crude protein level basis corresponding to the feed ingredient in an attempt to consider if challenge affects AA digestibility differently in individual ingredients and in mixture of ingredients akin to practical diets formulation. As such, SID values for Met in the corn, wheat, and SBM diets were not only lower than that of PM, CSP, and WSP diets, but numerically were more affected by the challenge. Meanwhile, the SID values of most indispensable and dispensable AA were affected by the diet and challenge interaction effect, suggesting that an *Eimeria* infection not only increases ITEF of AA but also that the efficiency of AA utilization in the body is governed by sufficient intake and availability of limiting AA. In this manner, it stands to reason why the SID values of the corn and wheat diets, were generally lower than that of the CSP and WSP values.

When comparing CSP and WSP diets, the analyzed chemical composition of the diets revealed comparable CP, and AA content, however, the ITEF and SID values between the two dietary treatments were quite varied and generally higher in the birds fed the CSP diet compared to the WSP diet. A similar trend was seen in a study by Persia et al. (2006), where AA digestibility values were lower in the *Eimeria*-challenged chicks fed a wheat-barley-pectin diet compared to *Eimeria*-challenged chicks fed a corn-SBM diet. This may be attributed to the differences in soluble arabinoxylans in wheat and corn (Williams, 2005: Kiarie et al., 2014; Sanchez et al., 2021). It has been suggested that the higher amount of soluble non-starch polysaccharides found in wheat, can increase digesta viscosity which escalates GIT transit and promotes the proliferation of certain undesirable microbes such as *Clostridium perfringens*, the causative agent for necrotic enteritis (Dal Pont et al., 2020). In combination with the intestinal damage caused by coccidiosis, excess mucin production as a consequence to the replication of sporozoites within the mucosal epithelium provides a beneficial source of nutrients for *C. perfringens* proliferation, thus further exacerbating the inflammation and disruption of the gut and negatively impacting digestive and absorptive capacities (Adhikari et al., 2020).

Moreover, the functional role of a particular AA may influence whether an AA may become limiting in response to an enteric disturbance such as coccidiosis. In a study by Faure et al. (2006), Thr, Ser, Pro, and Cys supplementation was shown to potentially promote mucin synthesis in inflammatory situations, suggesting that these AA play a role in mucosal healing. Correspondingly, a significant decrease in SID of Thr, Pro, and Ser in challenged birds (61.1, 56.6, and 40.6% difference in response to challenge, respectively was observed across all dietary treatments in the current study. When looking at the BEL, the largest increases in endogenous loss were seen for Asp, Pro, Lys, and Thr. Both Pro and Thr are commonly found in intestinal and pancreatic secretions, as well as mucin (Ravindran, 2016). Therefore, it is logical to see a significant decrease in SID of Thr and Pro as these AA are highly amenable to changes in metabolic conditions that precipitates endogenous losses (Adedokun et al., 2011).

The lack of challenge response in SID of Met, Asp, and Cys was surprising when the functional role of these AA is considered. Sulfur-containing AA such as Met and Cys, can be metabolized into glutathione, homocysteine, and taurine, all of which play major roles in defending cells against immune and oxidative stress (Yang and Liao, 2019). While Asp is interconvertible with oxaloacetate, an intermediate of the tricarboxylic acid (**TCA**) cycle which is the central component of cellular metabolism (Alpers, 2001). However, Gautier and Rochell (2020) evaluated the impact of a coccidiosis vaccine on AID of AA in broilers fed corn-, soybean meal-, and distillers dried grains with solubles (**DDGS**)-based diets and showed no response to vaccination for AID of Met, Asp, and Cys. There was a general reduction in AID of AA in corn diets, a general increase in AID of AA in DDGS diets, and minimal impact on SBM diets, thus the study concluded that the impact of the coccidiosis vaccination on AA digestibility varied depending on ingredient (Gautier and Rochell, 2020). According to the range of SID values of Met, Asp, and Cys reported in literature, the SID values from our nonchallenged birds were in general lower for Met in corn, wheat, and SBM, and higher for Cys in corn, wheat, SBM, and PM (Adedokun et al., 2007b, 2015; Ullah et al., 2016; Blok and Dekker, 2017). The SID of Asp values from our nonchallenged birds were lower than the reported SID values in corn and wheat, while higher than the reported SID values in SBM and PM (Adedokun et al., 2007b, 2015; Ullah et al., 2016; Blok and Dekker, 2017). Most likely, these differences are associated with AA profile of ingredients and the capacity for AA utilization. With this said, the challenge response may have been affected by the limited supply of AA in relation to the ingredient and diet type (single ingredient compared to mixed ingredient).

Additionally, the birds we used in our study were challenged on d 10, a time at which the small intestine is still developing and undergoing morphological changes (Noy and Sklan, 1997; Sklan and Noy, 2003). Restriction of feed between the age of 7 and 20 d has been shown to influence growth and feed efficiency weeks later in chicks (Plavnik and Hurwitz, 1988; Noy and Sklan, 1997). Even though the birds were given the same basal starter diet initially, the change in nutrient supply from the experimental diets in addition to the *Eimeria* challenge introduced shortly thereafter, may have also affected digestion and absorption of certain AA. This may have been the case for SID of Lys from the nonchallenged birds fed the wheat diets, where the coefficient was much lower than the range of SID values reported in literature (Bandegan et al., 2011; Blok and Dekker, 2017).

In conclusion, the results of the present study demonstrated that both diet, challenge, and their interaction can all significantly influence ileal AA digestibility. While challenge affected the SID of AA of certain dietary treatments more than others, this may have been due to the AA profiles corresponding to the ingredient and the diet, which can influence *Eimeria* pathogenicity. *Eimeria* challenge had the largest impact on the SID of Thr, Pro and Ser across all dietary treatments, suggesting digestible content of these AA may be lower in broiler chickens exposed to *Eimeria*.

## Appendix Supplementary materials

Image, application 1
