# Supplementary material for: Standardized ileal digestibility of amino acids in broiler chickens fed single or mixture of feed ingredients-based diets with or without Eimeria challenge
Source: Poult Sci. 2022 Mar 10;101(6):101839. doi: 10.1016/j.psj.2022.101839 (PMC9018447; doi:10.1016/j.psj.2022.101839)
Supplement: Supplementary file 1 [file mmc1.docx]

**Supplementary table 1.** Plasma concentration (*μ*M) of indispensable and dispensable amino acids in broiler chickens fed nitrogen-free diet (NFD) without or with *Eimeria* challenge^1^

|  | NFD - | NFD + | SEM | Probabilities | |
| --- | --- | --- | --- | --- | --- |
| Indispensable |  | |  |  | |
| Arg | 162.50 | 124.00 | 19.25 | 0.174 |  |
| His | 138.00 | 120.00 | 9.00 | 0.492 |  |
| Ile | 74.83 | 66.17 | 4.33 | 0.514 |  |
| Leu | 136.00 | 137.00 | 0.50 | 0.964 |  |
| Lys | 479.83 | 497.50 | 8.84 | 0.893 |  |
| Met | 61.50 | 51.00 | 5.25 | 0.282 |  |
| Phe | 103.50 | 102.33 | 0.59 | 0.900 |  |
| Thr | 182.83 | 168.17 | 7.33 | 0.802 |  |
| Val | 44.00 | 47.33 | 1.67 | 0.298 |  |
| Mean | 194.17 | 166.50 | 13.84 | 0.733 |  |
|  |  |  |  |  |  |
| Dispensable |  |  |  |  |  |
| Ala | 449.83 | 505.33 | 27.75 | 0.686 |  |
| Asp | 36.50 | 45.50 | 4.50 | 0.527 |  |
| Cys | 60.50 | 58.67 | 0.91 | 0.907 |  |
| Glu | 130.33 | 139.17 | 4.42 | 0.630 |  |
| Gly | 526.33 | 443.50 | 41.42 | 0.483 |  |
| Pro | 213.17 | 231.16 | 9.00 | 0.644 |  |
| Ser | 1529.17 | 1282.17 | 123.50 | 0.245 |  |
| Tyr | 116.17^a^ | 76.83^b^ | 19.67 | 0.024 |  |
| Mean | 375.17 | 347.83 | 13.67 | 0.569 |  |

^1^ “-” unchallenged, “+” challenged

Within a row LSmeans assigned different superscripts differs, *P*<0.05**.**

**Supplementary table 2.** Apparent ileal digestibility (%) of indispensable amino acids in different feedstuff-based diets fed to broiler chickens with or without coccidiosis challenge

|  |  | Arg | His | Ile | Leu | Lys | Met | Phe | Thr | Val | Mean |
| --- | --- | --- | --- | --- | --- | --- | --- | --- | --- | --- | --- |
| Interaction effects | |  |  |  |  |  |  |  |  |  |  |
| Diet | *Eimeria*^1^ |  |  |  |  |  |  |  |  |  |  |
| Corn | - | 88.90^ab^ | 58.69 | 47.09 | 70.92 | 57.75 | 82.86 | 59.68 | 49.92^ef^ | 48.43^bcd^ | 62.69 |
| Wheat | - | 93.79^a^ | 60.57 | 58.35 | 64.95 | 21.36 | 69.09 | 66.85 | 61.38^bcde^ | 49.87^bc^ | 60.69 |
| SBM^2^ | - | 86.54^ab^ | 71.25 | 71.15 | 71.87 | 79.12 | 81.78 | 71.29 | 74.84^abc^ | 67.61^ab^ | 75.05 |
| PM^3^ | - | 77.04^abc^ | 78.29 | 76.22 | 78.52 | 79.86 | 88.76 | 78.90 | 88.47^a^ | 74.34^a^ | 80.05 |
| CSP^4^ | - | 87.08^ab^ | 81.01 | 79.59 | 82.85 | 84.21 | 91.46 | 81.54 | 76.06^abc^ | 78.11^a^ | 82.44 |
| WSP^5^ | - | 85.62^ab^ | 79.59 | 78.15 | 78.95 | 81.39 | 87.59 | 78.64 | 70.90^abcd^ | 75.37^a^ | 79.58 |
| Corn | + | 74.13^bc^ | 51.98 | 44.09 | 69.27 | 46.52 | 70.88 | 56.94 | 18.75^h^ | 44.94^cd^ | 53.06 |
| Wheat | + | 61.61^c^ | 25.18 | 34.83 | 41.73 | -16.23 | 59.80 | 47.43 | 40.42^fg^ | 27.28^d^ | 35.78 |
| SBM | + | 75.95^bc^ | 44.38 | 51.08 | 51.90 | 59.18 | 69.32 | 50.79 | 54.78^def^ | 40.45^cd^ | 55.31 |
| PM | + | 65.70^c^ | 49.34 | 60.37 | 62.41 | 63.60 | 90.19 | 62.82 | 79.28^ab^ | 57.23^abc^ | 65.66 |
| CSP | + | 73.42^bc^ | 62.76 | 63.14 | 66.50 | 68.83 | 90.67 | 63.71 | 56.45^cdef^ | 58.58^abc^ | 67.12 |
| WSP | + | 75.48^bc^ | 43.98 | 50.54 | 51.09 | 55.26 | 82.68 | 49.34 | 29.16^gh^ | 43.03^cd^ | 53.40 |
| SEM |  | 2.81 | 4.90 | 4.20 | 3.59 | 8.39 | 3.05 | 3.41 | 6.11 | 4.60 | 3.98 |
| Main effects of diet | |  |  |  |  |  |  |  |  |  |  |
|  | Corn | 81.52 | 55.34^bc^ | 45.59^b^ | 70.10^ab^ | 52.14^b^ | 76.87^ab^ | 58.31^b^ | 34.34 | 46.69 | 57.88^bc^ |
|  | Wheat | 77.70 | 42.88^c^ | 46.59^b^ | 53.34^c^ | 2.57^c^ | 64.45^b^ | 57.14^b^ | 50.90 | 38.58 | 48.24^c^ |
|  | SBM | 81.25 | 57.82^abc^ | 61.12^a^ | 61.89^bc^ | 69.15^ab^ | 75.55^ab^ | 61.04^ab^ | 64.81 | 54.03 | 65.18^ab^ |
|  | PM | 71.37 | 63.82^ab^ | 68.30^a^ | 70.47^ab^ | 71.73^a^ | 89.48^a^ | 70.86^a^ | 83.88 | 65.79 | 72.86^a^ |
|  | CSP | 80.25 | 71.89^a^ | 71.37^a^ | 74.68^a^ | 76.52^a^ | 91.07^a^ | 72.63^a^ | 66.26 | 68.35 | 74.78^a^ |
|  | WSP | 80.55 | 61.79^ab^ | 64.35^a^ | 65.02^abc^ | 68.33^ab^ | 85.14^a^ | 63.99^ab^ | 50.03 | 59.20 | 66.49^a^ |
| SEM |  | 1.58 | 3.97 | 4.49 | 3.11 | 11.34 | 4.12 | 2.64 | 6.97 | 4.66 | 4.03 |
| Main effects of *Eimeria* | |  |  |  |  |  |  |  |  |  |  |
| - |  | 86.50 | 71.57^a^ | 68.43^a^ | 74.68^a^ | 67.28^a^ | 83.59 | 72.82^a^ | 70.26 | 65.62 | 73.42^a^ |
| + |  | 71.05 | 46.27^b^ | 50.68^b^ | 57.15^b^ | 46.19^b^ | 77.26 | 55.17^b^ | 46.47 | 45.25 | 55.06^b^ |
| SEM |  | 7.72 | 12.65 | 8.88 | 8.76 | 10.54 | 3.17 | 8.82 | 11.89 | 10.19 | 9.18 |
| Probabilities |  |  |  |  |  |  |  |  |  |  |  |
| Diet |  | 0.031 | <0.001 | <0.001 | 0.001 | <0.001 | 0.001 | 0.001 | <0.001 | <0.001 | <0.001 |
| *Eimeria* |  | <0.001 | <0.001 | <0.001 | <0.001 | <0.001 | 0.094 | <0.001 | <0.001 | <0.001 | <0.001 |
| Diet x *Eimeria* |  | 0.020 | 0.068 | 0.190 | 0.073 | 0.322 | 0.839 | 0.059 | 0.007 | 0.049 | 0.217 |

N=6

^1^“-” unchallenged, “+” challenged

^2^Soybean meal

^3^Pork meal

^4^Corn-soybean meal-pork meal

^5^Wheat-soybean meal-pork meal

Means assigned different letters within a response criterion are significantly different, *P* < 0.05.

**Supplementary table 3.** Apparent ileal digestibility (%) of dispensable amino acids in different feedstuff-based diets fed to broiler chickens with or without coccidiosis challenge

|  |  | Ala | Asp | Cys | Glu | Gly | Pro | Ser | Tyr | Mean |
| --- | --- | --- | --- | --- | --- | --- | --- | --- | --- | --- |
| Interaction effects | |  |  |  |  |  |  |  |  |  |
| Diet | *Eimeria*^1^ |  |  |  |  |  |  |  |  |  |
| Corn | - | 64.50 | 63.62 | 93.79 | 66.79^de^ | 73.64^ab^ | 69.59 | 54.13 | 56.03 | 67.76 |
| Wheat | - | 72.98 | 62.51 | 83.90 | 90.29^a^ | 79.62^a^ | 64.40 | 60.42 | 60.75 | 71.86 |
| SBM^2^ | - | 77.06 | 92.22 | 96.96 | 88.14^ab^ | 74.88^ab^ | 80.48 | 68.06 | 77.60 | 81.93 |
| PM^3^ | - | 36.05 | 87.82 | 87.82 | 46.75^f^ | 51.21^c^ | 81.05 | 74.78 | 56.12 | 65.20 |
| CSP^4^ | - | 85.01 | 79.55 | 97.10 | 90.05^a^ | 76.13^a^ | 83.41 | 78.06 | 76.54 | 83.23 |
| WSP^5^ | - | 73.62 | 76.35 | 97.33 | 91.11^a^ | 69.99^ab^ | 84.69 | 74.41 | 84.92 | 81.55 |
| Corn | + | 52.73 | 55.75 | 86.77 | 62.82^e^ | 68.25^abc^ | 51.85 | 44.33 | 52.76 | 59.41 |
| Wheat | + | 51.10 | 65.39 | 82.06 | 78.77^abcd^ | 79.69^a^ | 33.24 | 26.23 | 64.68 | 60.14 |
| SBM | + | 70.28 | 86.67 | 92.79 | 76.35^bcd^ | 58.32^bc^ | 51.74 | 44.29 | 66.68 | 68.39 |
| PM | + | 34.40 | 79.76 | 86.06 | 16.99^g^ | 17.97^d^ | 65.42 | 43.77 | 48.72 | 49.14 |
| CSP | + | 80.64 | 73.10 | 95.31 | 81.12^abc^ | 58.38^bc^ | 69.04 | 61.67 | 60.85 | 72.52 |
| WSP | + | 69.12 | 69.39 | 93.98 | 73.76^cde^ | 65.02^abc^ | 57.61 | 42.63 | 71.88 | 67.92 |
| SEM |  | 4.83 | 3.28 | 1.59 | 6.28 | 4.96 | 4.49 | 4.66 | 3.20 | 2.93 |
| Main effects of diet | |  |  |  |  |  |  |  |  |  |
|  | Corn | 58.62^c^ | 59.69^e^ | 90.28^abc^ | 64.81 | 70.95 | 60.72^bc^ | 49.23^b^ | 54.40^bc^ | 63.59^bc^ |
|  | Wheat | 62.04^bc^ | 63.95^de^ | 82.98^c^ | 84.53 | 79.66 | 48.82^c^ | 43.33^b^ | 62.72^abc^ | 66.00^b^ |
|  | SBM | 73.67^ab^ | 89.45^a^ | 94.88^ab^ | 82.25 | 66.60 | 66.11^ab^ | 56.18^ab^ | 72.14^ab^ | 75.16^a^ |
|  | PM | 35.23^d^ | 83.79^ab^ | 86.94^bc^ | 31.87 | 34.59 | 73.24^a^ | 59.28^ab^ | 52.42^c^ | 57.17^c^ |
|  | CSP | 82.83^a^ | 76.33^bc^ | 96.21^a^ | 85.59 | 67.26 | 76.23^a^ | 69.87^a^ | 68.70^abc^ | 77.88^a^ |
|  | WSP | 71.37^ab^ | 72.87^cd^ | 95.66^a^ | 82.44 | 67.51 | 71.15^ab^ | 58.52^ab^ | 78.40^a^ | 74.74^a^ |
| SEM |  | 6.74 | 4.64 | 2.20 | 8.60 | 6.29 | 4.11 | 3.72 | 4.16 | 3.31 |
| Main effects of *Eimeria* | |  |  |  |  |  |  |  |  |  |
| - |  | 68.20^a^ | 77.01^a^ | 92.82 | 78.86 | 70.91 | 77.27^a^ | 68.31^a^ | 68.66^a^ | 75.26^a^ |
| + |  | 59.71^b^ | 71.68^b^ | 89.50 | 64.97 | 57.94 | 54.82^b^ | 43.82^b^ | 60.93^b^ | 62.92^b^ |
| SEM |  | 4.25 | 2.67 | 1.66 | 6.94 | 6.49 | 11.23 | 12.25 | 3.87 | 6.17 |
| Probabilities |  |  |  |  |  |  |  |  |  |  |
| Diet |  | <0.001 | <0.001 | <0.001 | <0.001 | <0.001 | <0.001 | 0.001 | 0.001 | <0.001 |
| *Eimeria* |  | 0.001 | 0.013 | 0.058 | <0.001 | <0.001 | <0.001 | <0.001 | 0.048 | <0.001 |
| Diet x *Eimeria* |  | 0.160 | 0.687 | 0.941 | 0.001 | 0.001 | 0.210 | 0.225 | 0.724 | 0.530 |

N=6

^1^“-” unchallenged, “+” challenged

^2^Soybean meal

^3^Pork meal

^4^Corn-soybean meal-pork meal

^5^Wheat-soybean meal-pork meal

Means assigned different letters within a response criterion are significantly different, *P* < 0.05.
